# Design and assembly of the 117-kb *Phaeodactylum tricornutum* chloroplast genome

**DOI:** 10.1093/plphys/kiad670

**Published:** 2023-12-19

**Authors:** Emma J L Walker, Mark Pampuch, Nelson Chang, Ryan R Cochrane, Bogumil J Karas

**Affiliations:** Department of Biochemistry, Schulich School of Medicine and Dentistry, Western University, London, ON N6A 5C1, Canada; Department of Biochemistry, Schulich School of Medicine and Dentistry, Western University, London, ON N6A 5C1, Canada; Department of Biochemistry, Schulich School of Medicine and Dentistry, Western University, London, ON N6A 5C1, Canada; Department of Biochemistry, Schulich School of Medicine and Dentistry, Western University, London, ON N6A 5C1, Canada; Department of Biochemistry, Schulich School of Medicine and Dentistry, Western University, London, ON N6A 5C1, Canada

## Abstract

There is growing impetus to expand the repertoire of chassis available to synthetic biologists. Chloroplast genomes present an interesting alternative for engineering photosynthetic eukaryotes; however, development of the chloroplast as a synthetic biology chassis has been limited by a lack of efficient techniques for whole-genome cloning and engineering. Here, we demonstrate two approaches for cloning the 117-kb *Phaeodactylum tricornutum* chloroplast genome that have 90% to 100% efficiency when screening as few as 10 yeast (*Saccharomyces cerevisiae*) colonies following yeast assembly. The first method reconstitutes the genome from PCR-amplified fragments, whereas the second method involves precloning these fragments into individual plasmids from which they can later be released. In both cases, overlapping fragments of the chloroplast genome and a cloning vector are homologously recombined into a singular contig through yeast assembly. The cloned chloroplast genome can be stably maintained and propagated within *Escherichia coli*, which provides an exciting opportunity for engineering a delivery mechanism for bringing DNA directly to the algal chloroplast. Also, one of the cloned genomes was designed to contain a single *Sap*I site within the yeast *URA3* (coding for orotidine-5′-phosphate decarboxylase) open-reading frame, which can be used to linearize the genome and integrate designer cassettes via golden-gate cloning or further iterations of yeast assembly. The methods presented here could be extrapolated to other species—particularly those with a similar chloroplast genome size and architecture (e.g. *Thalassiosira pseudonana*).

## Introduction

Having the tools to clone, deliver, and install whole genomes provides the utmost potential for engineering an organism to meet any biologically possible desire. Historically, the subjects of such grand transformations have been limited to relatively simple or otherwise extensively studied microorganisms (e.g. viruses (Cello et al. 2002; Chan et al. 2005), bacteria (Gibson et al. 2010; Fredens et al. 2019), and yeast (Annaluru et al. 2014)). There is a growing impetus to diversify the repertoire of synthetic biology chassis capable of such large-scale genome engineering feats. Of particular interest is the development of a chassis with photosynthetic capabilities that could serve as a platform for the sustainable generation of bioproducts.

Chloroplast genomes could function as unique chassis for engineering photosynthetic eukaryotes. From an assembly perspective, the reduced size of the chloroplast genome (e.g. 110 to 160 kb) and its “nakedness” (i.e. lack of histones) (Bock 2015) makes constructing it from synthetic DNA more feasible than that of most nuclear chromosomes. Several other characteristics make it an industrially attractive target for encoding an array of bioproducts (e.g. medicines (Chan et al. 2016; Hoelscher et al. 2018), biofuels (Li et al. 2018; Richter et al. 2018), and bio-fertilizers (Ivleva et al. 2016; Eseverri et al. 2020)). In contrast to the nuclear counterpart, the chloroplast genome can be site-specifically modified through innate homologous recombination processes, thereby avoiding integration into transcriptionally inactive regions of the genome (i.e. position effects), and lacks gene silencing machinery that could otherwise lead to postinsertion transgene repression (Bock 2015). Furthermore, the chloroplast microenvironment is enveloped by at least two lipid bilayers. This enables the spatial compartmentalization of transgenic pathways, which can increase their efficiency, and the containment of recombinant proteins or metabolic by-products, which could be otherwise deleterious if present in the cytosol (Huttanus and Feng 2017). These characteristics have enabled some chloroplast-expressed transgenes to comprise 2% to 25% of total soluble protein (TSP) levels within photosynthetic plant cells (Meyers et al. 2010), though there are records of transgenes exceeding 70% TSP levels (Oey et al. 2009). In comparison, nuclear expression systems often produce 1% to 2% TSP levels within the same systems (Meyers et al. 2010).

The development of the chloroplast as a synthetic biology chassis has been limited by a lack of efficient techniques for whole-genome cloning and engineering. To the best of our knowledge, there are only two published papers demonstrating the potential for cloning an entire chloroplast genome outside of the organelle. The first, which was published in 1991, details the capture of the maize (*Zea mays*) chloroplast genome in a yeast artificial chromosome (YAC) (Gupta and Hoo 1991). Here, *Z. mays* chloroplast DNA was fragmented with an endonuclease, ligated into YACs, and then transformed into yeast (*Saccharomyces cerevisiae*), generating a YAC library for the chloroplast genome (Gupta and Hoo 1991). More than 10,000 yeast transformants were screened, with only one putative clone containing what diagnostically appeared to be the whole chloroplast genome (Gupta and Hoo 1991). Over two decades later, O’Neill et al. (2011) made use of an existing bacterial artificial chromosome (BAC) library to clone the entire *Chlamydomonas reinhardtii* chloroplast genome. For this, six BACs containing overlapping fragments of the chloroplast genome were modified to contain various yeast and bacterial cloning elements before being assembled into a singular contig through homologous recombination in yeast (O’Neill et al. 2011). This strategy was more efficient than the previous, with 3 out of 30 yeast transformants demonstrating the correctly assembled whole genome, but it depended upon the preexistence of a characterized BAC library (O’Neill et al. 2011). BAC/YAC libraries do not exist for all photosynthetic eukaryotes, and creating these libraries is technically demanding and time consuming (Jansen et al. 2005). As well, relying upon BAC/YAC libraries does not permit the utmost design flexibility for assembling large constructs; the user is limited to whichever fragments are captured during the library preparation stage, which is determined by the DNA shearing method employed (e.g. enzymatic digestion, physical shearing) and can be, resultingly, indiscriminate. An efficient and tractable assembly strategy for rapidly generating whole chloroplast genomes is a necessary first step to achieve the full potential of this chassis.

We sought to design and test alternative assembly strategies for cloning whole chloroplast genomes. We chose *Phaeodactylum tricornutum* as our model organism due to its ease of propagation and rapidly growing toolbox for genetic engineering, characteristics that have made it the prospect of a synthetic genome project (Pampuch et al. 2022). The preceding study demonstrated that the chloroplast genome of *C. reinhardtii* (34.57% G + C) could be stably maintained in yeast and *Escherichia coli* (O’Neill et al. 2011); however, the stability and maintenance of the *P. tricornutum* chloroplast genome (32.15% G + C) has yet to be explored. This genome could pose unexpected challenges when cloning in *E. coli* (∼50% G + C) because sequences with high A + T content are more likely to contain spurious open reading frames and/or origins of replication, which can cause genome toxicity and instability, respectively (Godiska et al. 2005).

In this report, we present a PCR-based and precloned assembly strategy for cloning the *P. tricornutum* chloroplast genome. Our methods demonstrated 90% to 100% efficiency when screening as few as 10 yeast colonies and three *E. coli* colonies following whole-genome assembly and transformation, respectively. The cloned genomes were able to be stably maintained in *E. coli* for 60 generations and did not pose a noticeable growth burden when maintained at a single-copy number. A combination of the assembly approaches was used to generate a genome containing a single *Sap*I site that can be used as a landing pad for integrating transgenic cassettes. We believe this efficient and tractable method for creating designer chloroplast genomes could be extrapolated to other photosynthetic eukaryotes, particularly those with similar genome sizes and architectures.

## Results

### Cloning of the *P. tricornutum* chloroplast genome

**Figure 1.**
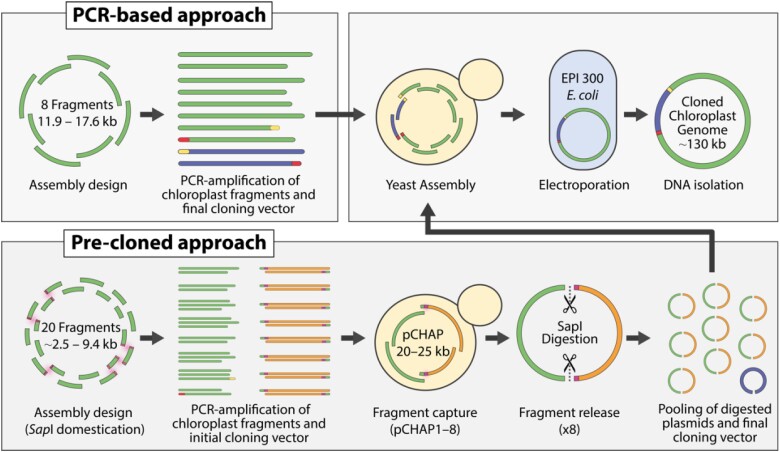
The two strategies for assembling the 117 kb chloroplast genome. The approaches differ in how the chloroplast fragments for whole-genome assembly are obtained. Endogenous and engineered *Sap*I restriction sites are highlighted in the precloned approach.

We developed two methods for cloning the chloroplast genome, both of which ultimately use yeast assembly to reconstitute the genome from eight overlapping fragments. The first method relies entirely on PCR-derived fragments for assembly and is aptly named the PCR-based approach (Fig. 1, top panel). The second method relies upon the cloning of PCR-derived fragments into individual plasmids that they can later be released from, this was termed the precloned approach (Fig. 1, bottom panel). The PCR-based approach was first attempted as this enabled us to rapidly test various assembly setups by simply designing different sets of primers. This resulted in an efficient assembly design that was used to inform the precloned approach, which requires more time and energy to initially establish, but permits greater downstream flexibility once generated. For instance, if we wanted to recode a region of the chloroplast genome, we could commercially synthesize the respective fragment and assemble it alongside the other precloned regions of the genome. Additionally, plasmids generated through the precloned approach can be easily shared between institutions (deposited on Addgene, Supplementary Table S1).

### Design strategy for partitioning the genome

It was first necessary to partition the 117 kb chloroplast genome into fragments that could be readily PCR-amplified. Most chloroplast genomes possess two inverted repeat regions—denoted as IRa and IRb—that can vary in size and, when present, bisect the genome into a large single copy (LSC) and a small single copy (SSC) (Bock 2015). As well, most chloroplast genomes are highly compact and contain very few noncoding regions (Bock 2015). In consideration of these characteristics, we partitioned the genome according to the following principles: (i) the inverted repeat regions (6,912 bp) were flanked on either end by at least 1,000 bp of nonrepetitive DNA to avoid unwanted homologous recombination when attempting assembly of the whole chloroplast genome, (ii) fragment termini overlapped by at least 40 bp, but ideally within the range of 100 to 300 bp, to facilitate homologous recombination, and (iii) the final cloning vector was inserted into a noncoding region of the genome. The last design consideration is premeditative, as we hope to one day transform the cloned genome back into *P. tricornutum*; inserting the vector into a coding region would be more likely to perturb the genome's function in vivo. Other design elements were considered depending on the objectives of the assembly strategy, as described in the proceeding sections.

### PCR-based assembly of the genome

**Figure 2.**
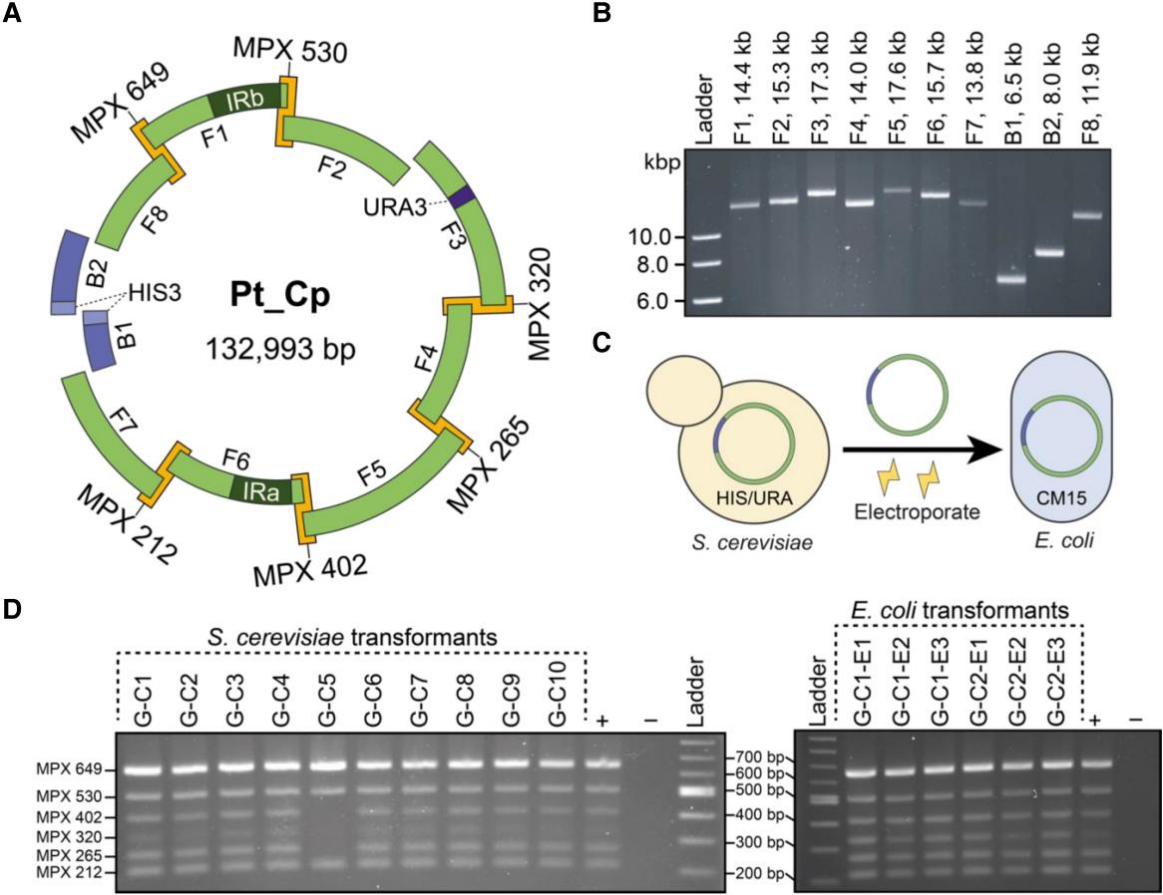
The assembly design for the PCR-based cloning method. **A)** The 10-fragment assembly includes the cloning vector pPt0521S_URA, which was split into two fragments at the *HIS3* marker (labeled B1 and B2), and the eight chloroplast fragments (labeled F1 to F8). Chloroplast fragment 3 was precloned to have a second yeast selection marker, *URA3*, in a noncoding region of the genome. MPX primers were designed to amplify six of the eight junctions between chloroplast fragments. **B)** The PCR-amplified fragments used for whole-genome assembly. **C)** Plasmids from candidate *S. cerevisiae* colonies were electroporated into electrocompetent *E. coli*. **D)** Screening of 10 yeast colonies following assembly of the whole genome. All but colony G-C5 screened positively. Plasmids from yeast colonies G-C1 and G-C2 were electroporated into *E. coli*, with all six transformants screening positively.

We first partitioned the 117 kb chloroplast genome into fragments that could be obtained through PCR amplification. Genome fragments ranged in size from 11.9 to 17.6 kb and overlapped by 100 to 300 bp at all junctions except for the site where the cloning vector would integrate (Fig. 2, A and B, Supplementary Table S2). Components of the pCC1BAC-derived cloning vector pPt0521S_URA were used for capturing the whole genome, which contains *HIS3* (coding for imidazoleglycerol-phosphate dehydratase) and chloramphenicol-resistance markers as well as other necessary elements for plasmid maintenance and propagation in *S. cerevisiae* and *E. coli* (GenBank: KP745602.1, Karas et al. 2015). We designed primers for integrating pPt0521S_URA into different regions of the genome and found that the noncoding region between hypothetical chloroplast open reading frame 88 (*f88)* and ribosomal protein CL22 (*rpl22)*, which maps to the junction between chloroplast fragments seven and eight (Supplementary Table S2), was the most easily amplifiable. For this, the cloning vector was amplified using 80-bp primers that would add ∼50 bp of overlaps for fragments seven and eight to its termini (Supplementary Table S3). To reduce the occurrence of false positive transformants during yeast assembly, the cloning vector was amplified as two overlapping fragments split at the yeast *HIS3* marker (Fig. 2A).

After a few failed attempts, we also integrated the yeast selective marker *URA3* into one of the chloroplast fragments to increase the probability of correct assembly of the whole chloroplast genome. The *URA3* marker was amplified using 80 bp primers that would add 40 bp of homologous sequences at both termini to a noncoding region in chloroplast fragment 3 (Supplementary Table S3). The third fragment was resultantly split into three smaller fragments, annotated as fragments 3A, 3B, and 3C (Supplementary Table S3, Supplementary Fig. S1). A 13-fragment assembly was performed in *S. cerevisiae* and demonstrated that the whole chloroplast genome could be recovered when using two yeast selective markers interspersed throughout the genome. To reduce the number of fragments needed for assembly, thereby increasing efficiency, the *URA3* marker was precloned into chloroplast fragment 3, generating the plasmid pCHAP3_URA (Supplementary Fig. S1). From this plasmid, the third fragment containing the integrated marker could be amplified as an intact piece via PCR and used in a 10-fragment assembly to reconstitute the whole genome.

The 10-fragment PCR-based assembly demonstrated that nine out of ten of *S. cerevisiae* colonies screened positively, as per the multiplex (MPX) PCR, when selected at random (Fig. 2D). When we electroporated the plasmids from two candidate *S. cerevisiae* colonies into *E. coli* (Fig. 2C), all the analyzed transformants screened positively (Fig. 2D). Plasmid DNA was isolated from *E. coli* colony G-C1-E1 and G-C2-E1 and sequenced, revealing that the whole chloroplast genome had been successfully captured through this method.

### Precloned approach for assembling the genome

The design strategy identified through the PCR-based approach was used to inform the precloned approach. Here, we first PCR amplified the chloroplast genome as 20 overlapping fragments ranging from 2.5 to 9.4 kb in length (Supplementary Table S3). Fragment termini were strategically placed at the six endogenous *Sap*I recognition sites (RSs) so that primers could be used to introduce silent mutations, thereby domesticating the chloroplast genome for *Sap*I (Supplementary Fig. S2A, Supplementary Table S4). Pairs or triads of fragments were then assembled with the cloning vector pSAP, a domesticated version of the cloning vector pCCBAC1_LC_TRP (GenBank: MN982904.1). The plasmids were designed such that the chloroplast fragments could be scarlessly released from their respective plasmids upon digestion with *Sap*I (Supplementary Fig. S2B). This ultimately generated eight unique plasmids, pCHAP1 to pCHAP8, that each harbored one of the eight overlapping chloroplast fragments identified through the PCR-based approach.

**Figure 3.**
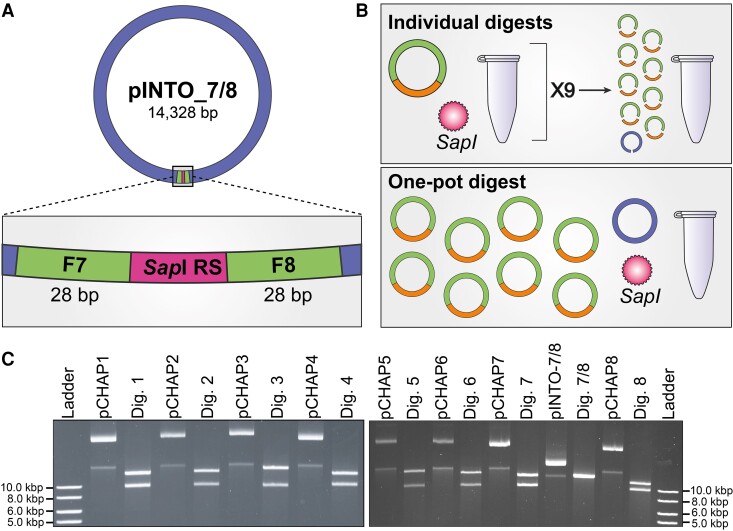
The precloned approach for assembly of the *P. tricornutum* chloroplast genome. **A)** pINTO_7/8 was designed to have a single *Sap*I RS flanked by 28 bp of homologous sequences to chloroplast fragments 7 and 8. When digested with *Sap*I, pINTO_7/8 is linearized, exposing the homologous sequences necessary for assembly. **B)** Two assembly mechanisms were possible—one in which all the plasmids are individually digested and validated ahead of assembly, and another in which all the plasmids are digested simultaneously in a one-pot reaction. The latter is more time-effective but cannot be assayed to ensure digestion is complete. **C)** Plasmids pCHAP1 to pCHAP8 and pINTO_7/8 were digested until completion via *Sap*I, liberating the individual chloroplast fragments from the cloning vector pSAP as well as linearizing the final cloning vector.

We also domesticated the cloning vector pPt0521S_URA (GenBank: KP745602.1), generating the plasmid pINTO_Sap. This plasmid was then assembled to have a *Sap*I RS flanked by 28 bp of homologous sequences to chloroplast fragments seven and eight, generating pINTO_7/8 (Fig. 3A). When digested with *Sap*I, pINTO_7/8 becomes linearized, exposing the 28 bp of homologous sequences at its termini. Plasmids pCHAP7 and pCHAP8 were designed to contain 20 bp of homologous sequences to the respective pINTO_7/8 termini, making it such that in total, the fragments shared 48 bp of overlap at the respective termini. The eight pCHAP plasmids and cloning vector pINTO_7/8 were digested, both individually and in a one-pot reaction (Fig. 3B), before transformation into *S. cerevisiae* for assembly of the whole genome. The individual reactions were assayed on a gel to ensure digestion was complete (Fig. 3C).

The precloned assembly approach demonstrated that 9 out of 10 (individual digests, Supplementary Fig. S3A) and 10 out of 10 (one-pot digest, Supplementary Fig. S3B) *S. cerevisiae* colonies screened positively, as per the MPX PCR, when selected at random. Plasmids from two candidate *S. cerevisiae* colonies per assembly were electroporated into *E. coli*, whereupon all the analyzed transformants screened positively (Supplementary Fig. S3). Plasmid DNA from *E. coli* colonies S-C2-E1 and C-C1-E1 was isolated and then sequenced, revealing that the whole chloroplast genome had been successfully reconstituted using both setups for the precloned assembly method.

### A hybrid approach for assembling the genome

We used a combination of the PCR- and precloned approaches to create a version of cloned genome that contains a single *Sap*I site in the *URA3* marker. The cloning vector pINTO_7/8 and pCHAP plasmids 1 to 2 and 4 to 8 were digested with *Sap*I and combined with chloroplast fragment 3, which had been PCR amplified from the plasmid pCHAP3_URA (Supplementary Fig. S4). The hybrid assembly approach demonstrated that 9 out of 10 *S. cerevisiae* colonies screened positively, as per the MPX PCR, when selected at random (Supplementary Fig. S4). Plasmids from two candidate *S. cerevisiae* colonies per assembly were electroporated into *E. coli*, whereupon all the analyzed transformants screened positively (Supplementary Fig. S4). Plasmid DNA from *E. coli* colony P-C1-E1 was isolated and then sequenced, revealing that the whole chloroplast genome containing a single *Sap*I in *URA3* site had been created.

### Maintenance of *P. tricornutum* chloroplast plasmids in *E. coli*

**Figure 4.**
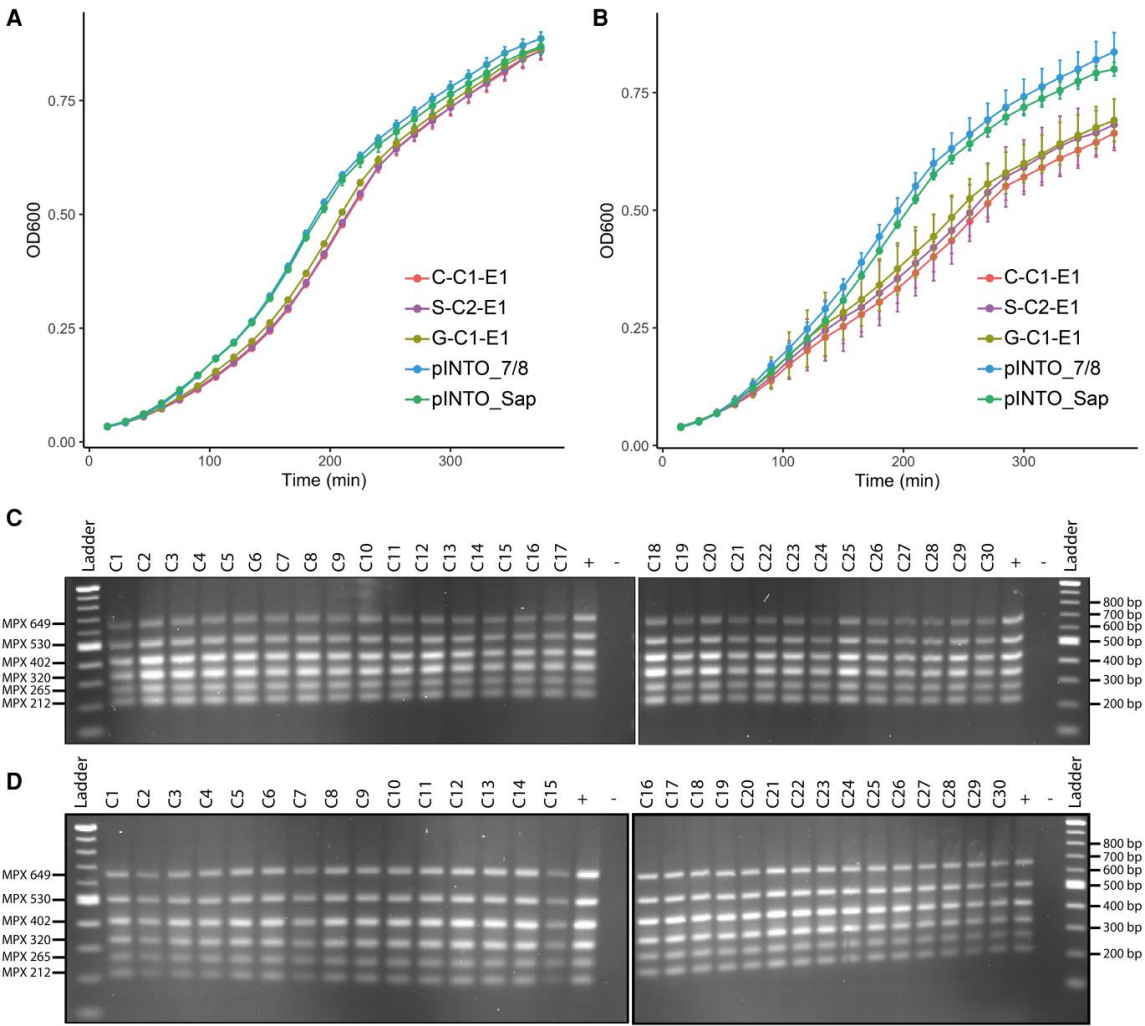
Analysis of the burden and stability of the cloned *P. tricornutum* chloroplast genome in *E. coli*. **A)** Under standard conditions (i.e. LB broth supplemented with CM15, 37 °C), *E. coli* strains harboring the chloroplast genome do not exhibit a growth deficit when compared to strains harboring smaller plasmids (∼14.3 kb, pINTO_7/8 and pINTO_Sap). **B)** When *E. coli* are induced to high-copy-number plasmid replication, strains harboring the chloroplast genome demonstrate a substantial growth burden. There were eight technical replicates per strain, with each point demonstrating the mean optical density for the measured time intervals. Errors bars demonstrate the standard deviation for the technical replicates at each time point. **C)** MPX screening of 30 colonies isolated from the *E. coli* strain harboring G-C1-E1 at time-point 0. All colonies demonstrate the expected banding pattern. **D)** MPX screening of 30 colonies isolated from the *E. coli* strain harboring G-C1-E1 at time-point 60 (i.e. 60 generations). All colonies demonstrate the expected banding pattern.

A growth assay was performed to determine if the cloned chloroplast genome poses a burden to *E. coli* (strain EPI300). Growth was measured in the presence and absence of arabinose, which, when present, induces high-copy number replication of the cloned chloroplast genome (∼132.9 kb). The growth rates of strains containing the chloroplast genome were measured against strains harboring pINTO_Sap and pINTO_7/8 (∼14.3 kb). Under noninduced conditions (i.e. no arabinose present), all strains demonstrate a similar growth rate (Fig. 4A); however, when plasmid replication is induced to high-copy number (i.e. arabinose present), the strains harboring the genome grow substantially slower and to a lower density than the strains harboring the comparatively small cloning vector (Fig. 4B).

Following this, a stability assay was conducted to see if the cloned genome can be stably maintained over the course of several bacterial generations. Thirty clones harboring the first-sequenced chloroplast genome (i.e. G-C1-E1) were grown for approximately 60 generations. DNA was isolated and screened from the clones prior to (Fig. 4C) and directly after (Fig. 4D) subculturing for this length of time. The MPX screen suggested that none of the clones had any discernable rearrangements or large-scale deletions within the plasmid, at least in the regions spanning six of the eight possible junctions between chloroplast fragments. To confirm this, the first three clones had their plasmids sequenced, demonstrating that the chloroplast genome had been maintained without any major rearrangements or deletions.

## Discussion

We have developed a strategy for whole-genome assembly that results in 90% to 100% efficiency when selecting as few as 10 *S. cerevisiae* colonies and three *E. coli* colonies following assembly and electroporation, respectively. Our strategy was first optimized using a PCR-based method, which allowed for the rapid testing of various assembly designs, before being adapted into the precloned approach. The latter assembly strategy enables greater downstream flexibility as the precloned fragments of the chloroplast genome can be individually manipulated or even commercially synthesized before attempting whole-genome assembly. As well, these plasmids can be readily shared between institutions through Addgene (Supplementary Table S4).

Initial attempts to assemble the genome in *S. cerevisiae* failed. This could have been due to recombination occurring between the inverted repeats (i.e. IRa, IRb) endogenously present within the chloroplast genome, or simply because of the size and number of fragments involved, which increased the complexity of the reaction. To generate greater selection pressure for the correct assembly, we incorporated a second yeast selective marker for the uracil biosynthetic pathway (i.e. *URA3*) into a noncoding region of the LSC. The second marker was positioned approximately 57.7 kb away from the cloning vector harboring *HIS3* in the SSC. The LSC and SSC are delineated by the inverse repeat regions; by positioning yeast selection markers on either side of this delineation, we aimed to encourage correct assembly of the SSC and LSC in *S. cerevisiae* when selecting on -HIS/URA media. This design proved successful at reconstituting the whole genome.

For the second assembly approach, fragments of the chloroplast genome were domesticated for endogenous *Sap*I sites and precloned into plasmids. The initial design for the whole genome was entirely domesticated for *Sap*I, which required domesticating the cloning vector pPt0521S_URA and the integrated *URA3* marker as well. However, the endogenous *Sap*I restriction site in *URA3* poses an interesting opportunity for engineering, and so a new design was created and assembled. Precloned and PCR-amplified fragments were combined to generate a cloned chloroplast genome that harbored a single *Sap*I site in the *URA3* marker. This restriction site can be used to integrate transgenic cassettes into the chloroplast genome through golden gate cloning or yeast assembly of the linearized plasmid and a transgenic fragment containing the appropriate overlaps (Supplementary Fig. S2C). The resulting transformed yeast can be selected for on -HIS media supplemented with 5-fluorotic acid (5-FOA). If *URA3* is still intact following assembly, 5-FOA will be metabolized into fluorouracil, thereby killing yeast with unsuccessfully assembled plasmids. This provides a simple and efficient method for rapidly integrating any transgenic cassette into the cloned chloroplast genome.

The cloned genomes were able to be stably maintained in *E. coli* and only perturbed growth rates when induced to high-copy number replication via supplementation of the media with arabinose. The cloning of partial- or whole-prokaryotic-derived genomes can cause issues within *E. coli* due to the unwanted expression of genes, which can pose energetic costs or even toxicity to the cell, or through competition for DNA replication and partitioning machinery between endogenous and introduced genetic material (Godiska et al. 2005). Another stability concern was that the *P. tricornutum* chloroplast genome has low G + C content (32.5%) relative to the *E. coli* genome (50.8%). Plasmids that are A + T rich can be challenging to clone in *E. coli* as these regions may spuriously form genetic elements like promoters, replication origins, open reading frames, and more (Godiska et al. 2005). In this instance, we believe that the decrease in growth rate under the induced growing condition is likely due to the increased energy-demand associated with replicating several copies (e.g. 10 to 100) of a large plasmid. The stability and replicability of the chloroplast genome under noninduced growing conditions presents a potential mechanism for delivering the cloned genome to *P. tricornutum* via bacterial conjugation (Karas et al. 2015). However, it is thought that plasmids are specifically targeted to the eukaryotic-cell nucleus during this conjugation; further engineering efforts will be required to realize the potential for conjugation as a transformation method for organelle engineering. In the meantime, the chloroplast genome could be transformed into *P. tricornutum* through biolistic bombardment or PEG-mediated transformation, which have been used to introduce foreign DNA into the chloroplasts of other microalgal species.

Before attempting to deliver the chloroplast genome through conjugation or any other transformation method, it will be necessary to disrupt recombinase A (*RecA)* and incorporate a chloroplast-specific selection marker into the cloned genome. The bacterial-derived *RecA* (PHATRDRAFT_54013) is expressed in the nuclear genome but localizes to the chloroplast after translation, where it mediates homologous recombination. Previous studies have demonstrated that disrupting this gene in a variety of distantly related photosynthetic eukaryotes disrupts homologous recombination within the chloroplast (Cerutti et al. 1995; Jeon et al. 2013). This will be necessary to prevent homologous recombination between the endogenous and cloned genomes, which posed challenges during transformation of the *C. reinhardtii* chloroplast genome (O’Neill et al. 2011). Many chloroplast-specific markers have been demonstrated in photosynthetic eukaryotes, but to-date, only the chloramphenicol-resistance marker, chloramphenicol acetyltransferase (*cat*) has been explored in *P. tricornutum* (Xie et al. 2014). It will be worthwhile to explore other resistance markers, like the commonly used streptomycin-resistance marker aminoglycoside adenyltransferase (*aadA*), to expand the organelle engineering toolbox for *P. tricornutum*.

Past attempts at cloning whole chloroplast genomes have relied upon the use of BAC/YAC libraries (Gupta and Hoo 1991; O’Neill et al. 2011), which are time-consuming to create and do not permit the utmost design flexibility when reconstructing the genome from overlapping fragments. Here, we present a design strategy for partitioning the 117 kb *P. tricornutum* chloroplast genome into overlapping fragments using PCR. These fragments can then be directly used for assembly with a cloning vector, or cloned separately into individual plasmids that they can be later released from. Given that the size and structure of the *P. tricornutum* chloroplast genome is similar to other microalgae species (e.g. *Thalassiosira pseudonana* [129 kb], *Odontella sinensis* [120 kb], *Guillardia theta* [122 kb] (Oudot-Le Secq et al. 2007)), we believe the design strategy presented in this paper could be used for the cloning of a myriad of chloroplast genomes.

**Figure 5.**
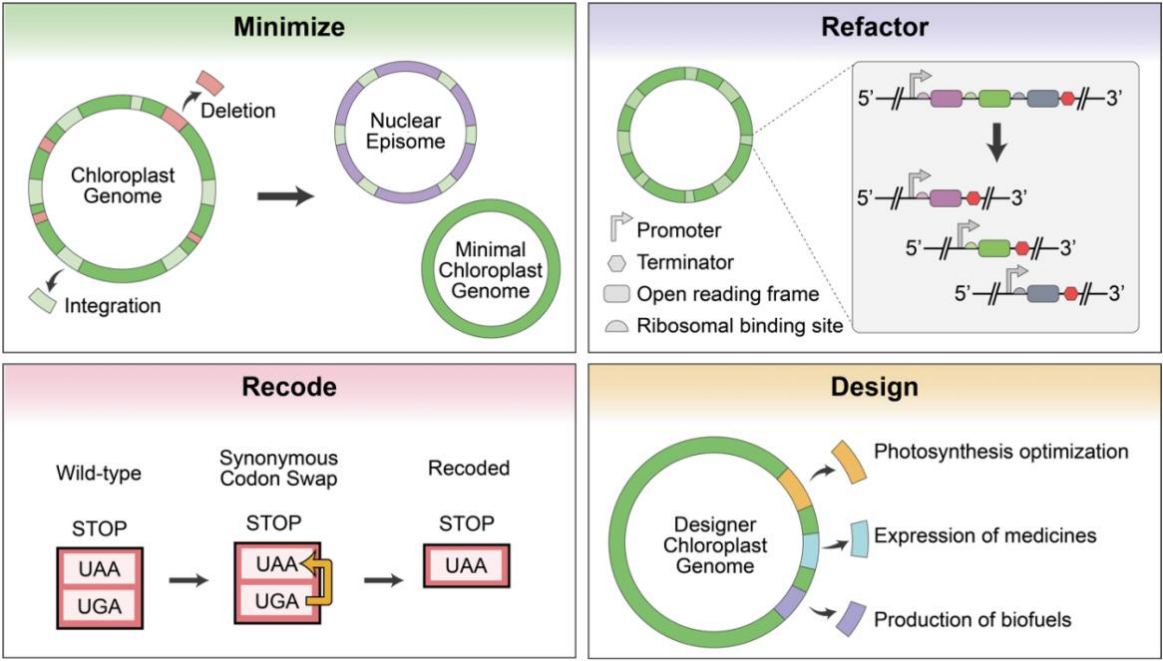
Potential applications for future designer chloroplast genomes (adapted from Fig. 6 of Esvelt and Wang (2013)). Genome minimization can be achieved by deleting nonessential regions of the genome and integrating essential genes into an episome for nuclear expression. Tractability can be increased by refactoring operons so that every gene has a unique promoter and terminator. Recoding of the stop codon UGA to UAA could facilitate codon reassignment to a non-natural amino acid for biocontainment purposes. Taken altogether with the proposed methods, it will be possible to design the chloroplast genome for several purposes.

In summary, we developed two approaches for efficient assembly of the *P. tricornutum* chloroplast genome and a facile mechanism for integration of transgenic cassettes, enabling the rapid generation of designer genomes. The chloroplast genome was able to be stably maintained and propagated in *E. coli* when plasmid replication was maintained at a low-copy number. To realize the full potential of this assembly pipeline, it will be necessary to develop methods for delivering, selecting for, and maintaining the cloned genome inside the recipient cell's chloroplast. The design-build cycle described herein could be adapted to generate *P. tricornutum* chloroplast genomes of any biologically conceivable imagination (Fig. 5) or even extrapolated to clone the chloroplast genomes of other photosynthetic eukaryotes.

## Materials and methods

### Strain and growth conditions

Yeast (*S. cerevisiae*) strain VL6-48 (genotype: *ATCC MYA-3666: MATα his3-Δ200 trp1-Δ1 ura3-52 lys2 ade2-1 met14 cir*^0^) was grown in 2X YPAD media (20 g L^−1^ yeast extract, 40 g L^−1^ peptone, 40 g L^−1^ glucose, and 160 mg L^−1^ adenine hemisulfate) or synthetic complete media (Teknova Inc.) supplemented with adenine (80 mg L^−1^) and lacking histidine (Cat #: C7112), tryptophan (Cat #: C7131), or both histidine and uracil (Cat #: C7221). Liquid yeast cultures were maintained at 30 °C in an orbital shaker set to 225 rpm. Solid yeast cultures were grown on 2% agar (w/v) plates incubated at the same temperature. Following spheroplast transformation, yeast cells were plated on the respective drop-out media supplemented with 1 M d-sorbitol.

*E. coli* strain EPI300 (LGC Biosearch Technologies, Cat #: EC02T110) was grown at 37 °C in Luria Broth (LB: 10 g L^−1^ tryptone, 5 g L^−1^ yeast extract, 10 g L^−1^ NaCl) supplemented with 15 *μ*g mL^−1^ chloramphenicol. Under induced conditions, cultures were grown for 5 to 6 h in LB that was supplemented with 15 *μ*g mL^−1^ chloramphenicol and 100 *μ*g mL^−1^ of L-(+)-arabinose. For colony screening, EPI300 transformants were plated on LB supplemented with 1.5% agar (w/v) and 15 *μ*g mL^−1^ chloramphenicol.

*P. tricornutum* (Culture Collection of Algae and Protozoa CCAP 1055/1) was grown in L1 media and maintained in a growth chamber at 18 °C under cool fluorescent lights (75 *μ*E m^−2^ s^−1^) for 16 h light/8 h dark conditions. L1 was made as previously described (Karas et al. 2015).

### Isolation, preparation, and sequencing of DNA

#### Isolation of DNA from *P. tricornutum*

*P. tricornutum* genomic DNA (high molecular weight) was obtained through phenol:chloroform:isoamyl alcohol isolation, as previously described (Giguere et al. 2022).

#### Isolation of DNA from *S. cerevisiae* and *E. coli* for transformation and PCR screening

Plasmids assembled in yeast and transformed into *E. coli* were isolated using a modified alkaline lysis protocol adapted from Karas et al. (2015). The process differs slightly between the organisms for the first few steps, as detailed below.

*S. cerevisiae* single colonies that had been streaked on a 2% agar (w/v) HIS/URA plate were inoculated in 3 mL of -HIS/URA media and placed in an orbital shaker set to 30 °C and 225 rpm. Once the culture reached high-density growth (approx. 40 to 48 h), 1.5 to 3 mL of cells were pelleted at 3,000 × *g* for 5 min. The supernatant was discarded and the cells were resuspended in 250 *μ*L resuspension buffer, which contained 240 *μ*L P1 (Qiagen, Cat #: 19051), 5 *μ*L of 1.4 M β-mercaptoethanol, and 5 μL zymolyase solution (200 mg Zymolyase 20 T [BioShop Canada Inc., Cat #: 120491-1], 9 mL H_2_O, 1 mL 1 M Tris pH 7.5, 10 mL 50% glycerol (v/v), stored at −20 °C) and incubated at 37 °C for 60 min.

*E. coli* strains harboring the cloned chloroplast genome or precloned plasmids (pCHAP1 to pCHAP8, pINTO_7/8) were grown overnight (approx. 16 h) in LB supplemented with 15 *μ*g mL^−1^ chloramphenicol. Then, 1.5 to 3.0 mL of *E. coli* cells were pelleted at 4,000 × *g* for 5 min. The supernatant was discarded and cells were resuspended in 250 *μ*L of resuspension buffer P1 (Qiagen, Cat #: 19051) supplemented with an additional 1 *μ*L of RNase cocktail (Invitrogen, Cat #: AM2286).

The lysis, neutralization, washing, and elution steps were the same between both organisms. Following resuspension, 250 *μ*L of lysis buffer P2 (Qiagen, Cat #: 19052) was added and the samples were inverted four to six times to mix. The samples were incubated at room temperature for 5 min before adding 250 *μ*L of neutralization buffer P3 (Qiagen, Cat #: 19053). The samples were quickly shaken four to six times to ensure that neutralization was complete before being spun at 16,000 × *g* for 10 min. The supernatant (approx. 750 *μ*L) was removed and transferred to a clean tube, whereupon 750 *μ*L of ice-cold 100% isopropanol was added. Samples were inverted and optionally stored at −20 °C for 20 min before being spun at 16,000 × *g* for 10 min. The supernatant was removed and 500 *μ*L of ice-cold 70% ethanol (v/v) was added. Samples were inverted to mix and then spun at 16,000 × *g* for 5 min. The supernatant was removed and the pellet was dried via an aspirator before being resuspended in 10 to 30 *μ*L of elution buffer (10 mM Tris–HCl pH 8.5).

#### Isolation of DNA from *E. coli* for sequencing

The modified alkaline lysis protocol described above isolates shorn genomic DNA and RNA alongside desired plasmid DNA. To isolate better quality plasmid DNA for sequencing, we used the column-based QIAGEN Large-Construct Kit (Cat. #: 12462). Isolation was performed in accordance with the product's manual, with the exception of the exonuclease step, which was not carried out. As well, to increase the amount of DNA isolated, *E. coli* strains were induced for high-copy number replication of the cloned genome. Here, overnight cultures of the respective *E. coli* strains were diluted 100× into LB supplemented with 15 *μ*g mL^−1^ chloramphenicol and 100 *μ*g mL^−1^ arabinose. The cells were grown for 5 to 6 h in an orbital shaker set to 37 °C and 225 rpm.

#### PCR amplification and preparation of fragments for assembly

Fragments for plasmid and whole-genome assembly were amplified with GXL polymerase (Takara, Cat. #: R050A) using the rapid PCR protocol and the primers are listed in Supplementary Table S3. Fragments that were amplified from a plasmid template were treated with 10 units (0.5 *µ*L) of *DpnI* (New England Biolabs Ltd., Cat. #: R0176). Samples treated with DpnI were incubated at 37 °C for 30 min before deactivation at 80 °C for 20 min. After deactivation, fragments were column-purified using the EZ-10 Spin Column PCR Products Purification Kit (BioBasic Inc., Cat. #: BS363).

#### Preparation of DNA for the precloned and hybrid approach

The isolated plasmids used in the precloned approach (pCHAP1 to pCHAP8, pINTO_7/8) had to be digested prior to conducting yeast assembly. For this, the plasmids were either digested in individual reactions or in a one-pot combined reaction.

For the individual reactions, 8 *µ*L of plasmid DNA was mixed with 1 *µ*L of CutSmart buffer and 1 *µ*L of *SapI* (New England Biolabs Ltd., Cat # R0569S) and then incubated at 37 °C for 3 h. The reaction was inactivated by heating it to 65 °C for 20 min. Then, 1 *µ*L of each reaction was ran on a 1% agarose (w/v) gel to check for complete digestion. Samples that demonstrated remnants of undigested plasmid were treated with 0.5 to 1 *µ*L of *SapI* and exposed to the same digestion and inactivation conditions as before. Then, 4 *µ*L of each completely digested plasmid were combined into an Eppendorf tube for use in yeast assembly.

For the combined digestion, plasmid concentrations were measured via the Qubit 2.0 fluorometer. Then, equimolar amounts of each plasmid were combined to create a 50 *µ*L reaction mixture containing 40 *µ*L of DNA (approx. 50 *µ*g of each plasmid), 5 *µ*L of CutSmart buffer, and 5 *µ*L of *SapI*. The digestion was carried out under the same reaction and inactivation conditions as above, and could not be checked for completion prior to use in assembly.

For the hybrid approach, pCHAP plasmids 1 to 8 as well as pINTO_7/8 were *Sap*I-digested in individual reactions as described above. Then, chloroplast fragment 3 containing *URA3* with a *Sap*I site was PCR-amplified from the plasmid pCHAP3_URA. The digested plasmids and PCR-fragment were adjusted to be approximately equimolar.

#### Sequencing of DNA

Whole plasmids were sequenced using long-read sequencing technology from Oxford Nanopore (SNPsaurus LLC and Flow Genomics Inc.). Samples were prepared in accordance with the specifications outlined by the sequencing vendors, who also performed read filtering and assembly of the reads into a single contig.

#### Alignment and mutational analysis of sequenced genomes

Sequences were aligned to a reference genome containing the annotated chloroplast genome (Oudot-Le Secq et al. 2007), *URA3* insertion, and *Sap*I-domesticated cloning vector (pINTO_7/8). Alignment was performed in Geneious Prime (version 2023.2; Biomatters Ltd, Kearse et al. 2012) using the MAFFT plugin (version 7.490, Katoh and Standley 2013) with the default parameters (algorithm: auto, scoring matrix: 200PAM/k = 2, gap open penalty: 1.53, offset value: 0.123). Aligned sequences were assessed for any rearrangements, insertions, deletions, and point mutations. The FASTA files for all of the sequenced clones and the reference genome can be found in the supplementary file Supplementary Data Set 1.

### DNA assembly via homologous recombination in *S. cerevisiae*

#### Spheroplasting and transformation of *S. cerevisiae*

Yeast were spheroplasted and transformed as previously described (Karas et al. 2014); however, fragments of DNA were used in place of bacterial cells. Assembly mixtures were composed of equimolar amounts of the respective fragments to generate a total reaction volume of 40 *µ*L, with there being 50 to 400 *µ*g of each fragment and 950 to 2,000 *µ*g of DNA per assembly on average. Following transformation and recovery of the cells in 1 mL of SOC, 700 and 300 *µ*L of transformants per assembly were resuspended in 8 mL of the respective 2% agar (w/v) drop-out media that had been melted and then cooled to 55 °C. This mixture was poured onto 2% agar (w/v) plates containing 15 mL of the precooled drop-out media. Once dry, the plates were transferred to a 30 °C incubator and grown until colonies appeared, which typically occurred within 3 to 5 d. The number of yeast transformants observed per genome assembly can be viewed in Supplementary Table S5. Assemblies for plasmids pSAP1 and pCHAP1 to pCHAP8 were selected for on complete media lacking tryptophan (i.e. -TRP), whereas the assembly for the final cloning vector pINTO_7/8 was plated on complete media lacking histidine (i.e. -HIS). Assemblies for the whole chloroplast genome were plated on complete media lacking histidine and uracil (i.e. -HIS/URA).

### Electroporation of plasmids into *E. coli*

Plasmids were isolated from yeast colonies as described above. For each electroporation reaction, 1 to 2 *μ*L of isolated plasmid DNA was combined with 25 to 50 *μ*L of homemade electrocompetent EPI300 cells (derived from LGC Biosearch Technologies, Cat #: EC02T110) in a sterile Eppendorf tube. The mixture sat on ice for approximately 5 min before being pipetted up-and-down twice and then transferred to a sterile 2 mm electroporation cuvette. Cuvettes were pulsed at 2.5 kV with a capacitance of 25 *μ*F and resistance of 200 Ω (Gene Pulser Xcell System, BioRad), generating a time constant of 4.9 to 5.2 ms. Electroporated cells were resuspended in 1000 *μ*L of SOC media and then transferred to sterile Eppendorf tubes for recovery at 37 °C for approximately 1 h. After recovery, 100 and 700 *μ*L of cells were spread across two 1.5% agar (w/v) LB plates supplemented with 15 *μ*g mL^−1^ chloramphenicol, which were then transferred to a 37 °C incubator. Tens to hundreds of transformants appeared across the plates within 24 h of spreading the electroporated cells.

### Colony PCR screening of *S. cerevisiae* and *E. coli*

#### *S. cerevisiae* screening strategy

Following whole-genome assembly, individual colonies of *S. cerevisiae* were picked with a sterile pipette-tip and transferred into 3 mL of -HIS/URA media supplemented with 100 *μ*g mL^−1^ ampicillin. Cultures were grown at 30 °C for approximately 2 d and then lysed for DNA isolation, as described above. For each colony assayed, 1 *μ*L of isolated plasmid DNA was used as template in a MPX PCR reaction (Multiplex PCR Kit, Qiagen, Cat #: 206143) with six primer sets (Supplementary Table S3). The reactions were carried out according to the manufacturer's specifications. PCR products were visualized on a 2% agarose (w/v) gel stained with ethidium bromide.

#### *E. coli* screening strategy

Single *E. coli* colonies were picked with a sterile pipette-tip and transferred to 3 mL of LB supplemented with CM15. Cultures were grown overnight at 37 °C and then lysed for DNA isolation, as described above. The same MPX PCR workflow for *S. cerevisiae* was followed for *E. coli*.

### Maintenance and stability of the cloned genome in *E. coli*

#### *E. coli* growth in liquid media

Five strains of *E. coli*, three of which harbored the cloned genomes (G-C1-E1, S-C2-E1, C-C1-E1) and two control plasmids (pINTO_7/8, pINTO_Sap), were prepared and measured as previously described (Cochrane et al. 2020). Eight technical replicates were performed for each strain across the two conditions. Wells used as blanks were filled with 200 *μ*L of the respective media, for which there were also eight technical replicates per condition, thereby filling every well in the plate. The blank reading for the respective row and condition was subtracted from the OD readings for the respective strains. Then, the mean and standard deviation were calculated per strain across the eight technical replicates. These measurements were plotted using R, with error bars demonstrating the standard deviation between technical replicates.

#### Plasmid stability in *E. coli* grown overall several generations

An *E. coli* strain harboring the whole genome (G-C1-E1) was prepared for the plasmid stability assay, as previously described (Cochrane et al. 2020). For both time points assayed, 30 *E. coli* colonies were picked at random, had their DNA isolated through the modified alkaline lysis protocol, and were assayed using the colony screening methods described above.

## Supplementary Material

kiad670_Supplementary_Data

## Data Availability

The data underlying this article are available in the article and in its online supplementary material.

## Supplementary data

The following materials are available in the online version of this article.

**Supplementary Figure S1.** Assembly of pCHAP3_URA from PCR-amplified fragments.

**Supplementary Figure S2.** Domestication of the chloroplast genome for *Sap*I.

**Supplementary Figure S3.** Precloned approach for assembly of the *P. tricornutum* chloroplast genome.

**Supplementary Figure S4.** Hybrid approach for assembly of the *P. tricornutum* chloroplast genome to create pPt_Sap.

**Supplementary Table S1.** Addgene IDs for deposited plasmids from this study.

**Supplementary Table S2.** Position of the PCR-amplified chloroplast fragments and inserts relative to the *P. tricornutum* reference chloroplast genome.

**Supplementary Table S3.** List of primer pairs used throughout this work.

**Supplementary Table S4.** List of silent mutations intentionally introduced to domesticate the chloroplast genome for *Sap*I.

**Supplementary Table S5.** *S. cerevisiae* colony forming units following assembly of the whole chloroplast genome through yeast assembly.

**Supplementary Data Set 1.** FASTA files for all of the sequenced clones and the reference genome.
