# Supplementary material for: Design and assembly of the 117-kb Phaeodactylum tricornutum chloroplast genome
Source: Plant Physiol. 2023 Dec 19;194(4):2217–28. doi: 10.1093/plphys/kiad670 (PMC10980414; doi:10.1093/plphys/kiad670)
Supplement: kiad670_Supplementary_Data [file kiad670_supplementary_data.zip › PP2023RR00842R1_Supplemental_Material.pdf]

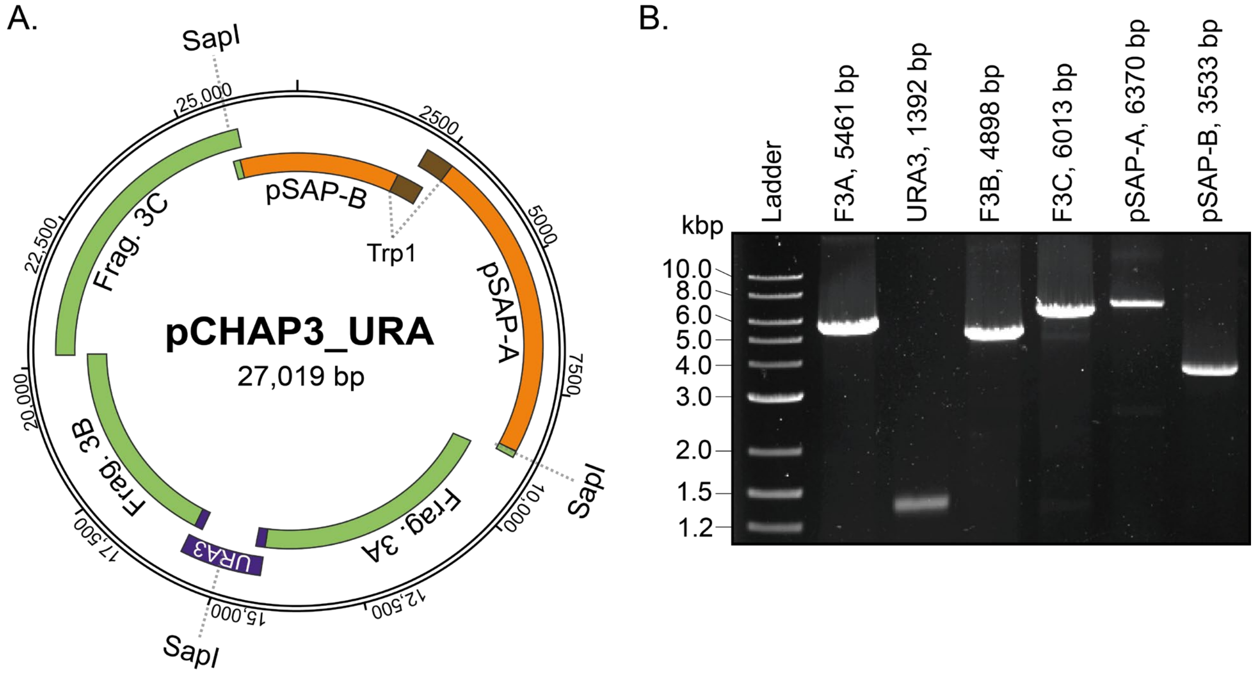

Supplemental Figure S1. Assembly of pCHAP3_URA from PCR-amplified fragments. **A.** Primers were used to add 35 bp of homology to the termini of the chloroplast fragments flanking the URA marker, 30 bp of homology to the termini of the pSAP fragments, and a *SapI* recognition site (8 bp). *URA*3 contains an endogenous *Sap*I site that was not domesticated in this assembly. **B.** The PCR-amplified fragments used for the assembly of pCHAP3_URA. Amplicons were visualized on a 1% agarose gel stained with ethidium bromide.

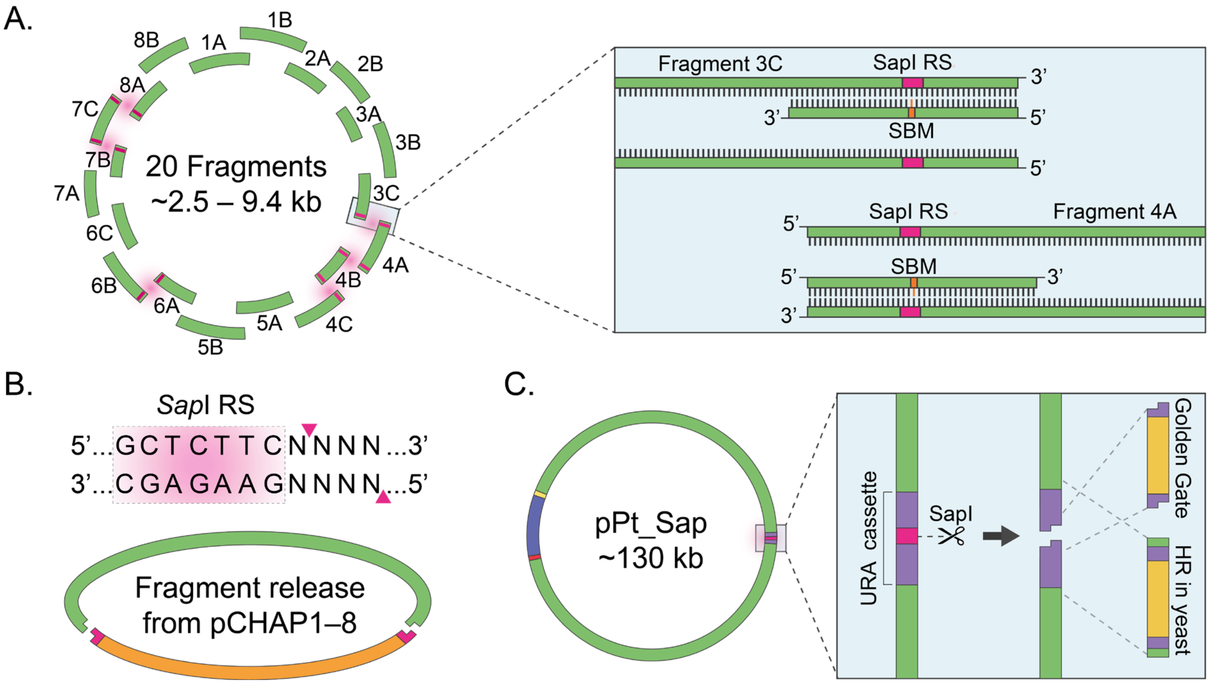

Supplemental Figure S2. Domestication of the chloroplast genome for *SapI*. **A.** Fragment termini were strategically placed near *SapI* recognition sites (RS, shown in magenta) so that primers could be designed to introduce silent mutations. **SBM**: single base pair mutation. **B**. *SapI* recognizes a 7 bp sequence (5’...GCTCTTC...3’) and cuts the adjacent non-specific base pairs, leaving a 5’ overhang of 3 bp. The pCHAP plasmids have been designed to scarlessly release individual chloroplast fragments upon digestion with *SapI*. **C**. DNA cassettes can be integrated into the designer chloroplast genome pPt_Sap via *in vitro* Golden Gate or *in vivo* yeast assembly. The termini of the DNA cassettes differ between approaches. **HR**: homologous recombination.

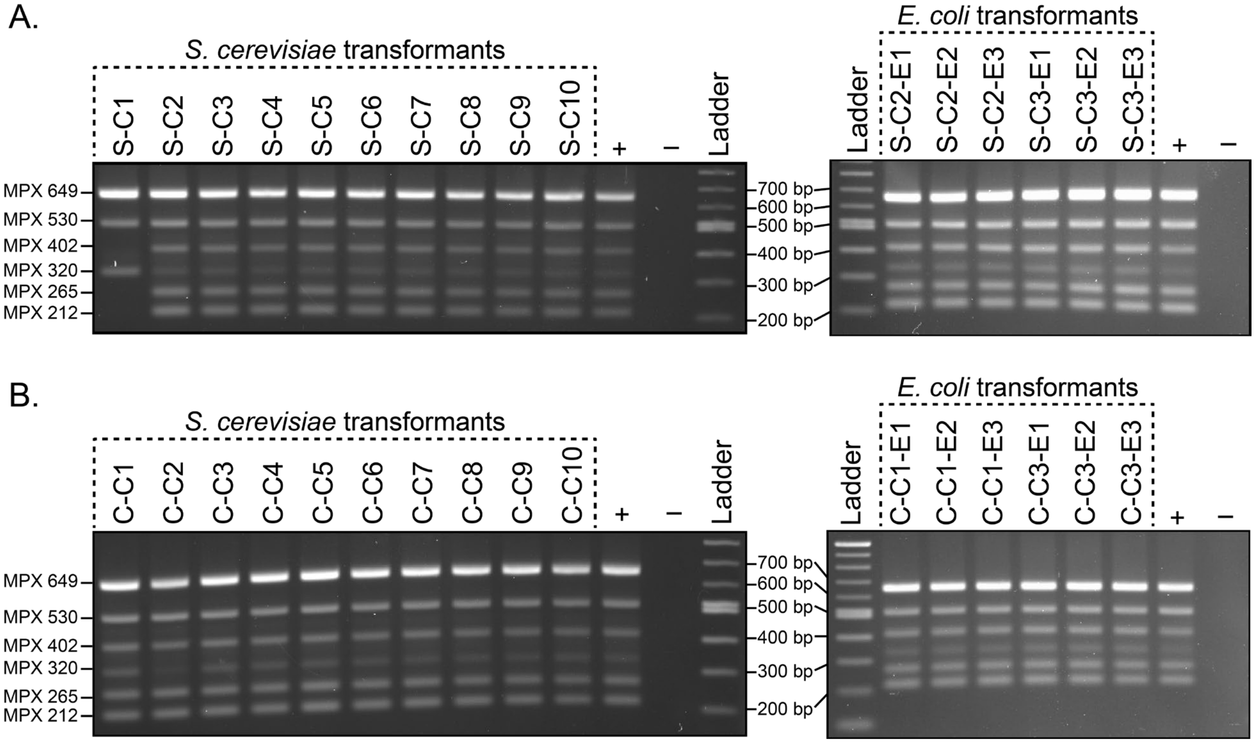

Supplemental Figure S3. Pre-cloned approach for assembly of the *P. tricornutum* chloroplast genome. **A**. Screening of ten yeast colonies following assembly of the whole genome from the individually digested plasmids. All but yeast colony S-C1 demonstrate the expected banding pattern. DNA from yeast colonies S-C2 and S-C3 was transformed into *E. coli*, with all transformants demonstrating the expected banding pattern. **B**. Screening of ten yeast colonies following assembly of the whole genome from the one-pot-digested plasmids. All colonies demonstrate the expected banding pattern. DNA from yeast colonies C-C1 and C-C2 was transformed into *E. coli*, with all transformants demonstrating the expected banding pattern. Amplicons were visualized on a 2% agarose gel stained with ethidium bromide.

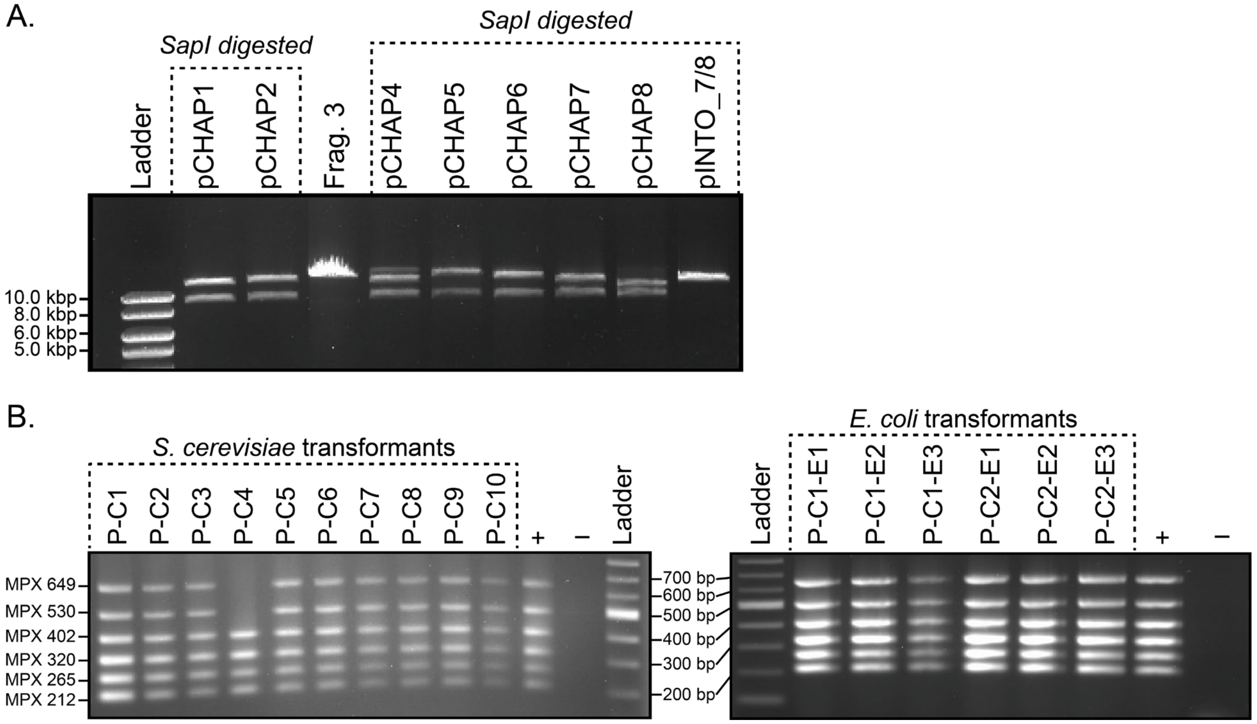

Supplemental Figure S4. Hybrid approach for assembly of the *P. tricornutum* chloroplast genome to create pPt_Sap. **A.** The plasmids pINTO_7/8 and pCHAP_1, 2, 4, 5, 6, 7, and 8 were digested with *Sap*I. Chloroplast fragment 3 was PCR-amplified from the plasmid pCHAP3_URA, which contains a single *Sap*I site located within the *URA3* coding region. **B**. Screening of ten yeast colonies following assembly of the whole genome using the individually digested plasmids and PCR-amplified fragment. All but yeast colony P-C4 demonstrate the expected banding pattern. DNA from yeast colonies P-C1 and P-C2 was transformed into *E. coli*, with all transformants demonstrating the expected banding pattern.

Supplemental Table S1. Addgene IDs for deposited plasmids from this study.

**Plasmid name**	**Addgene ID**
pCHAP1	206846
pCHAP2	206847
pCHAP3_URA	206848
pCHAP3_Sap	206849
pCHAP4	206850
pCHAP5	206851
pCHAP6	206852
pCHAP7	206853
pCHAP8	206857
pSAP	206429
pINTO_7/8	206431
pPt_Cp	206855
pPt_Sap	206856

Supplemental Table S2. Position of the PCR-amplified chloroplast fragments and inserts relative to the *P. tricornutum* reference chloroplast genome (GenBank accession: NC_008588, version: NC_008588.1, Oudot-Le Secq *et al.*, 2007). The position of fragments 3 and 4 differs between the PCR-based and pre-cloned approach, this was to position an endogenous *Sap*I site between the termini of these two fragments so that it could be domesticated using primers. Otherwise, the positioning of the fragments is the same between both approaches.

<table>
<tr><th colspan="2" rowspan="2">Fragment</th><th colspan="2">Location in the GenBank reference genome</th></tr>
<tr><th>From</th><th>To</th></tr>
<tr><td colspan="2">Genome fragment 1</td><td>103,415</td><td>568</td></tr>
<tr><td colspan="2">Genome fragment 2</td><td>302</td><td>15,550</td></tr>
<tr><td rowspan="2">Genome fragment 3</td><td>PCR-based approach fragment</td><td>15,300</td><td>30,873</td></tr>
<tr><td>Pre-cloned approach fragment</td><td>15,300</td><td>31,265</td></tr>
<tr><td colspan="2">URA3 insertion</td><td>20,723</td><td>20,724</td></tr>
<tr><td rowspan="2">Genome fragment 4</td><td>PCR-based approach fragment</td><td>30,639</td><td>45,214</td></tr>
<tr><td>Pre-cloned approach fragment</td><td>31,184</td><td>45,214</td></tr>
<tr><td colspan="2">Genome fragment 5</td><td>45,005</td><td>62,645</td></tr>
<tr><td colspan="2">Genome fragment 6</td><td>62,369</td><td>78,045</td></tr>
<tr><td colspan="2">Genome fragment 7</td><td>77,856</td><td>91,605</td></tr>
<tr><td colspan="2">Final cloning vector insertion</td><td>91,605</td><td>91,606</td></tr>
<tr><td colspan="2">Genome fragment 8</td><td>91,606</td><td>103,519</td></tr>
</table>

Supplemental Table S3. List of primer pairs used throughout this work. The highlighted base pairs (e.g., A, T, G, C) represent sites where a silent mutation was introduced to domesticate an endogenous *Sap*I restriction site.

**Amplicon**	**Primers (listed in 5′ to 3′ orientation)**	**Template DNA**	**Length (bp)**
Primers used to amplify fragments of the *P. tricornutum* chloroplast genome for assembly, used in the PCR-based approach (final design)			
Fragment 1	Forward: tggtgaaaacgttgaaatcg<br>Reverse: gtccaggtcgtggtggtact	Wild-type *P. tricornutum* genomic DNA	14,543
Fragment 2	Forward: acatccaagcccagactgat<br>Reverse: ttgttttcgttggttggtca		15,249
Fragment 3A	Forward: aagaccaccgatttgacaccaacgtgtgaaatcaccttgcgcttcagggccccataaaag<br>Reverse: ggcggacgcgatggatatgttctgccaagggcgcgccagtttttctggacttttcaagcc		5461
Fragment 3B	Forward: gtgcgcatgatcgtgctcctgtcgttgaggcgcgccagagtttttaacttctaaattaac<br>Reverse: tgtaaaaggtaagtactttaaaagtgtatcaatttgtactacaatgggaccatccgtaaa		4898
Fragment 3C	Forward: ttaaattctgtttttcttcaacttcttcaactacggctggagcagctgccattacaaca<br>Reverse: tgcacctgcgcgacatgaataaggtaattcaataccttgctc**C**tcagcagcatctaaaac		6013
Fragment 3 w/ URA3	Forward: aagaccaccgatttgacaccaacgtgtgaaatcaccttgcgcttcagggccccataaaag<br>Reverse: tgcacctgcgcgacatgaataaggtaattcaataccttgctc**C**tcagcagcatctaaaac	pCHAP3_URA	17,278
Fragment 4	Forward: tcatgttaaagttaaagactcaaaagg<br>Reverse: cgccatgttcagcaagtaaa	Wild-type *P. tricornutum* genomic DNA	14,576
Fragment 5	Forward: ggtttaaaacattagtgggtgga<br>Reverse: ccatgtgttgttccaacgag		17,691
Fragment 6	Forward: ttgctcgggtcttagctgat<br>Reverse: actggccggtttcattgtag		15,450
Fragment 7	Forward: tggaatttagttgggttacgc<br>Reverse: tcgtcggcaaaaaccttatc		14,092
Fragment 8	Forward: acattcgcatgtcacctcat<br>Reverse: ttatcaccggcaaaaccttc		11,780
Primers used to amplify the cloning vector, pPt0521S_URA, for integration between fragments 7 and 8, used in the PCR-based approach			
Fragment 1	Forward:<br>ttttgataaattaaattaaatgttcgtattaagtaaataattaacgaatttttatcttccgctgcataaccctgcttcgg<br>Reverse: ttcagtggtgtgatggtcgt	pPt0521S_URA	6516
Fragment 2	Forward: cagtagcagaacaggccaca<br>Reverse:<br>taaaaatttactgaaaaaaatcaaataaacttagagaaagagtaattcttaaaccaaagcggagtgactgc<br>aactaatga		8004
Primers used to generate pSAP, the domesticated cloning vector used to capture chloroplast fragments in the pre-cloned approach			
Fragment 1	Forward: atccggatgagaactgtttttgaacaggccattgatcaacgctcAtcaactggtgcctgg<br>Reverse: ggccgccagcccagcggcgagggcaaccaggggcttcgccctgtcgctcgactgcggcga	pCCBAC1_LC_<br>TRP	4784
Fragment 2	Forward: acgctagtgataataagtgactgaggtatgtgcGcttcttatctcctttttgtagtgttgc<br>Reverse: caggttcccaaatagaaagagcatttctccaggcaccagttgaTgagcgttgatcaatgg		1289
Fragment 3	Forward: gggggatcgccaacaaatactaccttttaccttgcTctacctgctctcaggtattaatgc<br>Reverse: aagttgtttaaaataagagcaacactacaaaaggagataagaagCgcacatacctcagtc		1561
Fragment 4	Forward: tcgccgcagtcgagcgacagggcgaagcccctggttgccctcgccgctgggctggcggcc<br>Reverse: aacaattcggcattaatacctgagagcaggTagagcaaggtaaaaggtagtatttgttgg		2395
Primers used to generate pCHAP1, the domesticated plasmid containing chloroplast fragment 1, used in the pre-cloned approach			
Fragment 1	Forward: tggtgaaaacgttgaaatcgtgggtgttaccgatacacaaacaacaacaattacgggtat<br>Reverse: ccgattggtgatgctaaagg	Wild-type *P. tricornutum* genomic DNA	6402
Fragment 2	Forward: agaatggcatttttgggtca<br>Reverse: gtccaggtcgtggtggtacttgtgatatttctgcttgggatgcattttatttagcaatgt		8369
Fragment 3	Forward: agaaatatcacaagtaccaccacgacctggactgaagagcctggttgccctcgccgctgg<br>Reverse: tgtctccacacctccgcttacatcaacaccaataacgccatttaatctaagcgcatcacc	pSAP	3533
Fragment 4	Forward: ttatgttagctggtggactgacgccagaaaatgttggtgatgcgcttagattaaatggcg		6370

	Reverse: cggtaacacccacgatttcaacgttttcaccatgaagagcgggcttcgccctgtcgctcg		
Primers used to generate pCHAP2, the domesticated plasmid containing chloroplast fragment 2, used in the pre-cloned approach			
Fragment 1	Forward: acatccaagcccagactgat<br>Reverse: atcacgtatcgcaaaccaca	Wild-type *P. tricornutum* genomic DNA	7288
Fragment 2	Forward: cgcatacgtgctcgatctaa<br>Reverse: ttgttttcgttggttggtca		8168
Fragment 3	Forward: taataataaacctgaccaaccaacgaaaacaatgaagagcctggttgccctcgccgctgg<br>Reverse: tgtctccacacctccgcttacatcaacaccaataacgccatttaatctaagcgcatcacc	pSAP	3533
Fragment 4	Forward: ttatgttagctggtggactgacgccagaaaatgttggtgatgcgcttagattaaatggcg<br>Reverse: tatgaataatttatcagtctgggcttggatgttgaagagcgggcttcgccctgtcgctcg		6370
Primers used to generate pCHAP3_URA, the plasmid containing chloroplast fragment 3, used in the pre-cloned approach<br>*SapI site in *URA3* is not domesticated in this plasmid			
Fragment 1	Forward: aagaccaccgatttgacaccaacgtgtgaaatcaccttgcgcttcagggccccataaaag<br>Reverse: ggcggacgcgatggatatgttctgccaagggcgcgccagtttttctggacttttcaagcc	Wild-type *P. tricornutum* genomic DNA	5461
URA3 ORF	Forward: aattttgtataccaataggcttgaaaagtccagaaaaactggcgcgcccttggcagaaca<br>Reverse: ttattactagaattacgttaatttagaagttaaaaactctggcgcgcctcaacgacagga	pTAMob	1392
Fragment 2	Forward: gtgcgcatgatcgtgctcctgtcgttgaggcgcgccagagtttttaacttctaaattaac<br>Reverse: tgtaaaaggtaagtactttaaaagtgtatcaatttgtactacaatgggaccatccgtaaa	Wild-type *P. tricornutum* genomic DNA	4898
Fragment 3	Forward: ttaaattctgtttttcttcaacttcttcaactacggctggagcagctgccattacaaca<br>Reverse: tgcacctgcgcgacatgaataaggtaattcaataccttgctcCtcagcagcatctaaaac		6013
Fragment 4	Forward: ttgaattaccttattcatgtcgcgcaggtgcatgaagagcctggttgccctcgccgctgg<br>Reverse: tgtctccacacctccgcttacatcaacaccaataacgccatttaatctaagcgcatcacc	pSAP	3533
Fragment 5	Forward: ttatgttagctggtggactgacgccagaaaatgttggtgatgcgcttagattaaatggcg<br>Reverse: atttcacacgttggtgtcaaatcggtggtctttgaagagcgggcttcgccctgtcgctcg		6370
Primers used to generate pCHAP3_SAP, the domesticated plasmid containing chloroplast fragment 3, used in the pre-cloned approach			
Fragment 1	Forward: aagaccaccgatttgacaccaacgtgtgaaatcaccttgcgcttcagggccccataaaag<br>Reverse: tctttgagcaataaagccgataacaaaatctttgtcgctTttcgcaatgtcaacagtacc	pCHAP3_URA	6279
Fragment 2	Forward: ctggagaatatactaagggtactgttgacattgcgaaAagcgacaaagattttgttatcg<br>Reverse: tgcacctgcgcgacatgaataaggtaattcaataccttgctcctcagcagcatctaaaac		11,076
Fragment 3	Forward: ttgaattaccttattcatgtcgcgcaggtgcatgaagagcctggttgccctcgccgctgg<br>Reverse: tgtctccacacctccgcttacatcaacaccaataacgccatttaatctaagcgcatcacc		3533
Fragment 4	Forward: ttatgttagctggtggactgacgccagaaaatgttggtgatgcgcttagattaaatggcg<br>Reverse: atttcacacgttggtgtcaaatcggtggtctttgaagagcgggcttcgccctgtcgctcg		6370
Primers used to generate pCHAP4, the domesticated plasmid containing chloroplast fragment 4, used in the pre-cloned approach			
Fragment 1	Forward: tgattgtaatgatgatgtatttgttttagatgctgctgaGgagcaaggtattgaattacc<br>Reverse: tagattcaacttcaacgatgttacaagtgggtattgttacagaagaAcattatgctacag	Wild-type *P. tricornutum* genomic DNA	2936
Fragment 2	Forward: gcgttgtaatgtttcttttacgttttctgctgtagcataatgTtcttctgtaacaatacc<br>Reverse: aaccaacaaaaacttattctttgcatggctaaaaaatctggGagagcattagtataattc		2979
Fragment 3	Forward: actaataaaaattatgaaaaaaaatatgaattatactaatgctctCccagattttttagc<br>Reverse: cgccatgttcagcaagtaaaatatttaattcacaatttactttataaacttcttcaccta		8292
Fragment 4	Forward: tgaattaaatattttacttgctgaacatggcgtgaagagcctggttgccctcgccgctgg<br>Reverse: tgtctccacacctccgcttacatcaacaccaataacgccatttaatctaagcgcatcacc	pSAP	3533
Fragment 5	Forward: ttatgttagctggtggactgacgccagaaaatgttggtgatgcgcttagattaaatggcg<br>Reverse: catctaaaacaaatacatcatcattacaatcatgaagagcgggcttcgccctgtcgctcg		6370
Primers used to generate pCHAP5, the domesticated plasmid containing chloroplast fragment 5, used in the pre-cloned approach			
Fragment 1	Forward: ggtttaaaacattagtgggtgga<br>Reverse: acctttcgcttccaattcct	Wild-type *P. tricornutum* genomic DNA	8559
Fragment 2	Forward: cagatttcttggccgaaatg<br>Reverse: ccatgtgttgttccaacgag		9398
Fragment 3	Forward: aaaggcgttcaactcgttggaacaacacatggtgaagagcctggttgccctcgccgctgg<br>Reverse: tgtctccacacctccgcttacatcaacaccaataacgccatttaatctaagcgcatcacc	pSAP	3533
Fragment 4	Forward: ttatgttagctggtggactgacgccagaaaatgttggtgatgcgcttagattaaatggcg<br>Reverse: gtaaactgttccacccactaatgttttaaacctgaagagcgggcttcgccctgtcgctcg		6370

Primers used to generate pCHAP6, the domesticated plasmid containing chloroplast fragment 6, used in the pre-cloned approach

Fragment 1	Forward: ttgctcgggtcttagctgat<br>Reverse: gcagccgaagatacaaaagc	Wild-type *P. tricornutum* genomic DNA	9379
Fragment 2	Forward: gcagttgttggtggtgaaat<br>Reverse: aaaaatttttgcgaacttccttcaaattgacttagaagtgaGgagctaaagatattttcc		2777
Fragment 3	Forward: tagttaaaaattttaaacggtcaaaaaaggaaaatatctttagctcCtcacttctaagtc<br>Reverse: actggccggtttcattgtag		3907
Fragment 4	Forward: atattgttcaatctacaatgaaaccggccagttgaagagcctggttgccctcgccgctgg<br>Reverse: tgtctccacacctccgcttacatcaacaccaataacgccatttaatctaagcgcatcacc	pSAP	3533
Fragment 5	Forward: ttatgttagctggtggactgacgccagaaaatgttggtgatgcgcttagattaaatggcg<br>Reverse: tttctccatttcatcagctaagacccgagcaatgaagagcgggcttcgccctgtcgctcg		6370

Primers used to generate pCHAP7, the domesticated plasmid containing chloroplast fragment 7, used in the plasmid-based approach
*Designed to contain an overlap for pIN_7/8

Fragment 1	Forward: tggaatttagttgggttacgctttaaaacaatggccaacgaaattattgagtatccatta<br>Reverse: gttatggaaaatacagcacgacgagatggacgtccaatcgaagaActtggctattatagc	Wild-type *P. tricornutum* genomic DNA	2490
Fragment 2	Forward: tacatcaaacttgtattgttttgtgattgggctataatagccaagTtcttcgattggacg<br>Reverse: aatttggcaattcgatcctg		5376
Fragment 3	Forward: cggggtttggtgaactaaga<br>Reverse:<br>accgaaaaaatcgctataatgaccccgaagcagggttatgcagcggaagataaaaattcgttaattatttacttaatacg		6274
Fragment 4	Forward: taacgaatttttatcttccgctgcataacccttgaagagcctggttgccctcgccgctgg<br>Reverse: tgtctccacacctccgcttacatcaacaccaataacgccatttaatctaagcgcatcacc	pSAP	3533
Fragment 5	Forward: ttatgttagctggtggactgacgccagaaaatgttggtgatgcgcttagattaaatggcg<br>Reverse: attgttttaaagcgtaacccaactaaattccaagaagagcgggcttcgccctgtcgctcg		6370

Primers used to generate pCHiP8, a semi-domesticated plasmid containing chloroplast fragment 8, a plasmid needed to generate pCHOP8
*Designed to contain an overlap for pINTO_7/8

Fragment 1	Forward: aatttaattttcattagttgcagtcactccgctttggtttaagaattactctttctctaagtttatttgatttttttcag<br>Reverse: ttcgattgatggtcgcataa	Wild-type *P. tricornutum* genomic DNA	5378
Fragment 2	Forward: tctcgacgcatttcaaacac<br>Reverse: ttatcaccggcaaaaccttcctctaaagttttttggaacatttcaatacccgtaattgtt		6845
Fragment 3	Forward: aaaactttagaggaaggttttgccggtgataatgaagagcctggttgccctcgccgctgg<br>Reverse: tgtctccacacctccgcttacatcaacaccaataacgccatttaatctaagcgcatcacc	pSAP	3533
Fragment 4	Forward: ttatgttagctggtggactgacgccagaaaatgttggtgatgcgcttagattaaatggcg<br>Reverse: agagtaattcttaaaccaaagcggagtgactgagaagagcgggcttcgccctgtcgctcg		6370

Primers used to generate pCHAP8, the domesticated plasmid containing chloroplast fragment 8, used in the pre-cloned approach
*Designed to contain an overlap for pINTO_Sap

Fragment 1	Forward: accaaagttaaagcgaattcgcccaagagctcaaggaagGgcatacaaaatattaaaacc<br>Reverse: ttcgattgatggtcgcataa	Wild-type *P. tricornutum* genomic DNA	5033
Fragment 2	Forward: tctcgacgcatttcaaacac<br>Reverse: ttatcaccggcaaaaccttcctctaaagttttttggaacatttcaatacccgtaattgtt		6845
Fragment 3	Forward: aaaactttagaggaaggttttgccggtgataatgaagagcctggttgccctcgccgctgg<br>Reverse: tgtctccacacctccgcttacatcaacaccaataacgccatttaatctaagcgcatcacc	pCHiP8	3533
Fragment 4	Forward: ttatgttagctggtggactgacgccagaaaatgttggtgatgcgcttagattaaatggcg<br>Reverse: cttcataaccactgtaatatgacaagtgggttttaatattttgtatgcCcttccttgagc		6797

Primers used to generate pINTO_Sap, a domesticated cloning vector that is needed to generate pINTO_7/8

Fragment 1	Forward: gcagcccacctatcaaggtgtactgccttccagacgaacgaaAagcgattgaggaaaagg<br>Reverse: aacaattcggcattaatacctgagagcaggaTgagcaaggtaaaaggtagtatttgttgg	pPt0521S_URA	5377
Fragment 2	Forward: gggggatcgccaacaaatactaccttttaccttgctcAtcctgctctcaggtattaatgc<br>Reverse: gagcaacactacaaaaggagataagaTgagcacatacctcagtcacttattatcactagc		1313
Fragment 3	Forward: acgctagtgataataagtgactgaggtatgtgctcAtcttatctccttttgtagtgttgc<br>Reverse: ggttcccaaatagaaagagcatttctccaggcaccagttgaagaAcgttgatcaatggcc		1287
Fragment 4	Forward: gaacaggccattgatcaacgTtcttcaactggtgcctggagaaatgctctttctatttgg<br>Reverse: ttttcctcaatcgctTttcgttcgtctggaaggcagtacaccttgataggtgggctgccc		6536

Primers used to generate pINTO_7/8, a domesticated cloning vector containing overlaps for chloroplast fragments 7 and 8

Fragment 1	Forward: ttctctaagtttattttgaagagcgctcttctgtattaagtaaataattaacgaatttttatcttccgctgcataaccct<br>Reverse: ggaagcggcggcggaagtcaggcatacgctggtaactttgaggcagctggtaacgctcta	pINTO_Sap	5005
Fragment 2	Forward: tacatatcaacccatatctcattcccttctttatcgggttacagaaccggtttacgcagt<br>Reverse: cctgctgcttaagtaattcctctttatctgtaaaggctttttgaagtgcatcacctgacc		4218
Fragment 3	Forward: gctggatgatgagcagatggctgcattatccagattgggtaacgattatcgcccaacaag<br>Reverse:<br>attatttacttaatacagaagagcgctcttcaaaataaacttagagaaagagtaattcttaaaccaaagcggagtgactg		5695
	Primers used in the multiplex screen for assaying colonies following whole genome assembly		
Junction between fragments 8 and 1	Forward: gtcccggatcagctttacaa<br>Reverse: cccgcttcaatagctacagg	DNA isolated for colony screens; see methods section	649
Junction between fragments 1 and 2	Forward: cggcagttgaagcaattaca<br>Reverse: gtccaggtcgtggtggtact		530
Junction between fragments 5 and 6	Forward: aacttgtcgagttggcagag<br>Reverse: ccatgtgttgttccaacgag		402
Junction between fragments 3 and 4	Forward: tcatgttaaagttaaagactcaaaagg<br>Reverse: tcgtagatagtatagtttcgctcgac		320
Junction between fragments 4 and 5	Forward: cggttatatttacaagcaacaagaaa<br>Reverse: cgccatgttcagcaagtaaa		265
Junction between fragments 6 and 7	Forward: tgtttactattatcaaacggattgga<br>Reverse: actggccggtttcattgtag		212

Supplemental Table S4. List of silent mutations intentionally introduced to domesticate the chloroplast genome for *Sap*I. The positions of the mutations are relative to the *P. tricornutum* reference chloroplast genome (GenBank accession: NC_008588, version: NC_008588.1, Oudot-Le Secq *et al.*, 2007).

Position	Strand	Mutation	Gene location	Effect
31,243	Forward	gaa → ga**g**	*petF*	glu → glu
34,093	Reverse	gag → ga**a**	*atpB*	glu → glu
36,988	Forward	ctt → ct**c**	*rpoB*	leu → leu
74,205	Forward	tct → tc**c**	*secA*	ser → ser
80,321	Reverse	gag → ga**a**	*rps16*	glu → glu
92,010	Forward	aga → ag**g**	*rpl22*	arg → arg

Supplemental Table S5. *S. cerevisiae* colony forming units following assembly of the whole chloroplast genome through yeast assembly.

Assembly approach	Volume of spheroplasts plated	
	700 µl	100 µl
PCR-based approach	361	38
Pre-cloned approach, individual digestion of plasmids	47	2
Pre-cloned approach, one-pot digestion of plasmids	15	5
Combinatory approach for assembling pPt_Sap	20	2
